# Foveal Thickness Fluctuations in Anti-VEGF Treatment for Central Retinal Vein Occlusion

**DOI:** 10.1016/j.xops.2023.100418

**Published:** 2023-10-29

**Authors:** Daisuke Nagasato, Yuki Muraoka, Mao Tanabe, Naomi Nishigori, Rie Osaka, Yoshinori Mitamura, Hitoshi Tabuchi, Tomoaki Murakami, Sotaro Ooto, Kiyoshi Suzuma, Akitaka Tsujikawa

**Affiliations:** 1 Department of Ophthalmology, Saneikai Tsukazaki Hospital, Himeji, Japan; 2 Department of Technology and Design Thinking for Medicine, Hiroshima University Graduate School, Hiroshima, Japan; 3 Department of Ophthalmology, Institute of Biomedical Sciences, Tokushima University Graduate School, Tokushima, Japan; 4 Department of Ophthalmology and Visual Sciences, Kyoto University Graduate School of Medicine, Kyoto, Japan; 5 Department of Ophthalmology, Kagawa University Faculty of Medicine, Kagawa, Japan

**Keywords:** Central retinal vein occlusion, Fluctuation, anti-VEGF

## Abstract

**Purpose:**

The aim of this study was to examine the effects of foveal thickness (FT) fluctuation (FTF) on 2-year visual and morphological outcomes of eyes with central retinal vein occlusion (CRVO) undergoing anti-VEGF treatment for recurrent macular edema (ME) based on a *pro re nata* regimen.

**Design:**

Retrospective, observational case series.

**Participants:**

We analyzed 141 treatment-naive patients (141 eyes) with CRVO-ME at a multicenter retinal practice.

**Methods:**

We assessed FT using OCT at each study visit. Patients were divided into groups 0, 1, 2, and 3 according to increasing FTF.

**Main Outcome Measures:**

We evaluated the logarithm of the minimal angle of resolution (logMAR) best-corrected visual acuity (BCVA), the length of the foveal ellipsoid zone (EZ) band defect measured using OCT, and the association of FTF with VA and EZ band defect length.

**Results:**

The mean baseline logMAR BCVA and FT were 0.65 ± 0.52 (Snellen equivalent range: 20/20–20/2000) and 661.1 ± 257.4 μm, respectively. The mean number of anti-VEGF injections administered was 5.6 ± 3.6. At the final examination, the mean logMAR BCVA and FT values were significantly improved relative to the baseline values (both *P* < 0.01). During the observation, BCVA longitudinally improved in Groups 0 and 1, remained unchanged in Group 2, and worsened in Group 3. Likewise, the length of the foveal EZ band defect did not increase in Group 0; however, it gradually increased in Groups 1, 2, and 3. Foveal thickness fluctuation was significantly and positively associated with the logMAR BCVA and length of the foveal EZ band defect at the final examination (*P* < 0.01). The final logMAR BCVA of patients developing neovascular complications was 1.27 ± 0.72 (Snellen equivalent range: 20/50–counting fingers), which was significantly poorer than that of patients without complications (*P* < 0.001). There was no significant difference in the neovascular complication rate among the FTF groups (*P* = 0.106, Fisher exact test).

**Conclusions:**

In eyes receiving anti-VEGF treatment for CRVO-ME, FTF can longitudinally impair the visual acuity and foveal photoreceptor status during the observation period, thus influencing the final outcomes. However, neovascular complications, which would also lead to a poor visual prognosis, may not be associated with FTF.

**Financial Disclosures:**

The authors have no proprietary or commercial interest in any materials discussed in this article.

Central retinal vein occlusion (CRVO) is a common retinal vascular disorder with a prevalence of approximately 1 in 1000.^1^ Macular edema (ME) is a major complication of CRVO, and it can decrease the visual acuity (VA) of the affected eye.^2^^,^^3^ In addition to ME, retinal ischemia and subsequent neovascular complications, namely vitreous hemorrhage (VH) and neovascular glaucoma (NVG), can lead to legal blindness in patients with CRVO. However, intravitreal injections of anti-VEGF agents have improved the visual and anatomical outcomes of patients with CRVO-ME.^4^, ^5^, ^6^, ^7^, ^8^ These improvements have been observed in both the short and long terms, with a significant improvement in the visual prognosis relative to that before anti-VEGF therapy.^9^

Additional injections of anti-VEGF agents based on a *pro re nata* (PRN) protocol is considered the clinical management strategy for recurrent CRVO-ME^10^, ^11^, ^12^; the usefulness of this protocol has been validated in several clinical trials.^13^^,^^14^ Central retinal vein occlusion-ME shows wide variations in each patient^15^; therefore, a fixed dosing protocol cannot always be applied.^9^^,^^16^ A PRN regimen helps in the management of such cases.

In previous studies of eyes receiving anti-VEGF therapies based on PRN protocols for age-related macular degeneration (AMD) and branch retinal vein occlusion (BRVO), eyes with greater foveal thickness (FT) fluctuation (FTF) showed poorer final VA values and more severe foveal damage than those with smaller fluctuations.^17^^,^^18^ Compared to eyes with BRVO, eyes with CRVO tend to have larger retinal nonperfusion areas that occasionally involve the macula, and they produce more intraocular VEGF. Retinal nonperfusion areas involving the macula are likely to resolve ME in eyes with CRVO.^19^ The effects of FTF on the visual and morphological outcomes of eyes receiving anti-VEGF therapy for CRVO-ME remain unclear. Therefore, the aim of the present study was to explore the effects of FTF on the VA, development of neovascular complications, and morphological outcomes in eyes with CRVO receiving long-term anti-VEGF treatment based on a PRN protocol for recurrent ME.

## Methods

This retrospective study adhered to the tenets of the Declaration of Helsinki and was approved by the ethics committees of Saneikai Tsukazaki Hospital (Hyogo, Japan), the Kyoto University Graduate School of Medicine (Kyoto, Japan), the Tokushima University Faculty of Medicine (Tokushima, Japan), and the Kagawa University Faculty of Medicine (Kagawa, Japan). The need for written informed consent for participation was waived because of the retrospective nature of the study. Instead, we created a home page presenting information on the purpose of the study for the subjects, and it was emphasized here that any subject could opt out of the study at any time via telephone, fax, or e-mail communication.

We retrospectively evaluated the data for patients with unilateral CRVO-ME who presented with symptoms for < 3 months before their initial treatment and visited one of the 4 aforementioned facilities between September 2013 and November 2016. At the initial visit, none of the patients had received any treatment for CRVO-ME. Other study inclusion criteria were a baseline FT of > 300 μm on OCT and a minimum follow-up period of 24 months from baseline. On the basis of these criteria, we evaluated 141 eyes of 141 consecutive patients presenting with unilateral, treatment-naive acute CRVO.

The affected eyes received intravitreal anti-VEGF injections of ranibizumab (Lucentis; 0.5 mg/0.05 mL, Novartis Pharma AG) or aflibercept (Eylea; 2.0 mg/0.05 mL, Bayer Pharma AG) for the treatment of ME and/or serous retinal detachment at the fovea according to the different regimens followed by each institute.

During the course of our study, none of the eyes received any other treatments for ME, such as bevacizumab injection, grid laser photocoagulation, steroid treatment, or surgical intervention. Subsequent to the initial injections at each facility, additional injections on a PRN basis were administered when ME or serous retinal detachment at the fovea was evident on OCT images, provided the FT was ≥ 300 μm and informed consent was obtained from the patient. We consistently used the same anti-VEGF agents for the initial and subsequent injections for each patient. Specific decisions regarding treatment schedules and injection intervals were taken at the discretion of each retinal specialist. However, patients were examined at least every 3 months in the follow-up period, as previously reported.^20^ It should be noted that the patient sample included in this study partially overlapped with that examined in our previous study.^9^

### Examinations

In each facility, retinal specialists diagnosed acute CRVO based on a medical interview regarding the onset of visual impairment as well as fundus examinations, which included slit-lamp biomicroscopy and OCT (Spectralis HRA + OCT, Heidelberg Engineering; RS-3000, Nidek; 3D OCT-1, Topcon). Different manufacturers and models of spectral domain-OCT instruments have varying performance characteristics, such as resolution, measurement speed, and signal-to-noise ratio. These variations can influence the measurement accuracy and reproducibility of the central retinal thickness, and different OCT instruments can yield differing central retinal thickness values.^21^ However, the reproducibility of measurements with the same instrument is typically high. The normative foveal retinal thickness is 265.1 ± 21.6 μm when measured by Spectralis HRA + OCT and 261.8 ± 20.0 μm when by measured by RS-3000. Consequently, during our study, FT of patients was consistently measured using the same OCT machine.

To assess the status of the retinal circulation, we performed ultrawidefield fluorescein angiography (FA; Optos 200Tx Imaging System, Optos PLC) for all patients. However, FA was not conducted on patients who had allergic reactions to the dye and those who did not provide consent for the examination, leading to 24 patients not undergoing FA evaluations at baseline. For patients who underwent initial FA, the procedure was repeated approximately 1 year after the commencement of anti-VEGF treatment or when symptoms such as a surge in fresh retinal hemorrhage, appearance of peripheral white vessels, or neovascular changes at the iris and/or retina were observed during routine ophthalmoscopic examinations. For clarity and consistency in assessing retinal nonperfusion areas, we selected FA images acquired 1 minute after dye injection, as these images are typically less affected by vascular leakage. Retinal specialists from each participating facility individually outlined these nonperfused areas and the disc area using the built-in OptosAdvance (Optos PLC) software. The nonperfusion area was then expressed in units of the averaged disc area. Cases were subsequently categorized as either nonischemic CRVO or ischemic CRVO following the methodology described in a prior report.^22^

At each facility, we assessed sex, age, smoking history, hypertension, dyslipidemia, diabetes mellitus, ischemic heart disease, and glaucoma at the initial visit. At each follow-up visit, we measured the best-corrected VA (BCVA) under standardized conditions using a standard Japanese Landolt VA chart, following an automatic refraction procedure performed with an autorefractometer to ensure accurate assessment of the refractive error and FT measured by OCT. Likewise, we measured the length of the foveal ellipsoid zone (EZ) band defect at each visit except the baseline visit. We also examined the status of the affected eye at baseline and at the 3-, 6-, and 12-month follow-up visits during the first year of the observation period.

**Figure 1:**
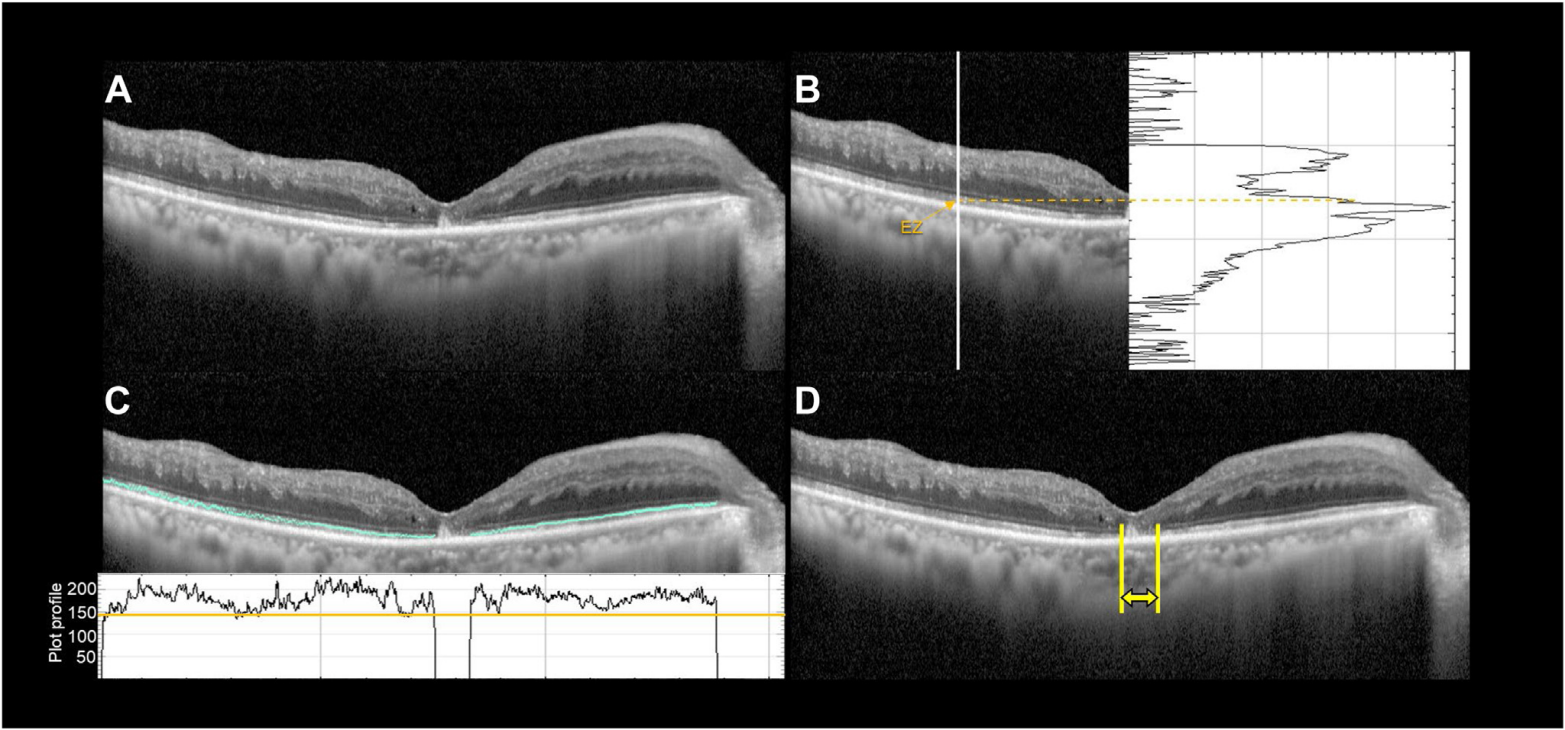
Procedure for measuring the foveal ellipsoid zone (EZ) band defect using spectral domain-OCT. **A,** Representative horizontal and vertical OCT B-scan images centered on the fovea. **B,** Defining the EZ band. The EZ band was identified by locating the point of highest reflectivity within the depth direction of the EZ layer, achieved through a plot profile. **C,** Defining the foveal EZ band defect. A defect was identified as a region within the central 2-mm area where the reflectivity fell 2 standard deviations below the region where the EZ band was readily evident. **D,** Process of measuring the length of the foveal EZ band defect. This measurement was derived from both the horizontal and vertical OCT B-scan images. The average value from both scans was considered the length of the EZ band defect.

In this study, we quantified the disruption in the foveal EZ band within a central 2-mm area on OCT images that horizontally and vertically bisected the center of the fovea. A single experienced retinal specialist at each facility assessed the signal intensity of the foveal EZ band on the OCT images using the plot profile function in ImageJ software (National Institutes of Health) in accordance with a previously reported method (Fig 1).^23^^,^^24^ Next, we calculated the average value of the lengths of the foveal EZ band defects on horizontal and vertical OCT sections. These lengths were truncated to an upper limit of 2000 μm at the measurement stage.

In this study, FT was measured on the OCT images of each eye according to a previously described method.^17^ More specifically, a thickness map of the whole retina was created using volume OCT scanning of the macula. Foveal thickness was defined as the average thickness within the central 1 mm subfield of the ETDRS grid, calculated as the mean distance between the vitreoretinal interface and the retinal pigment epithelium.

### Definition and Classification of FTF

To evaluate the degree of CRVO-ME recurrence during the observation period, we calculated the standard deviation (SD) in FT for each patient based on the FT values evaluated at each visit (except the baseline visit). Then, we evenly divided the included patients into 4 groups ranging from Group 0 (minimum SD) to Group 3 (maximum SD) in ascending order of SDs. The clinical parameters were compared among these groups. The SD ranges were 0 to 10.62, 10.62 to 57.08, 57.08 to 154.24, and 154.24 to 364.07 for Group 0, Group 1, Group 2, and Group 3, respectively. This methodology has been previously reported in investigations conducted among patients with AMD^17^ and BRVO.^18^

### Statistical Analysis

All data were statistically analyzed using Python Statsmodels (https://www.statsmodels.org), the scikit-learn package (https://scikit-learn.org/) and Py4Etrics (https://github.com/Py4Etrics/py4etrics). Data are presented as means ± SDs. Visual acuity measured using the Landolt chart were converted to logarithm of the minimum angle of resolution (logMAR) units.

We performed multiple regression analyses with a linear model in which the final logMAR BCVA was set as the objective variable. Age, baseline logMAR BCVA, baseline FT, and FTF were set as explanatory variables based on confirmation that there were no multicollinearities among these explanatory variables. Among the explanatory variables, continuous variables were standardized to a mean of 0 and a variance of 1. We also performed multiple comparisons of the final BCVA, final length of the foveal EZ band defect, follow-up duration, and number of anti-VEGF injections among groups; Tukey honest significant difference test was used for comparisons of the former 2 parameters while the Steel–Dwass test was used for comparisons of the latter parameter and the number of VH or NVG episodes. This study focused on the relationship between FTF and VA. In the previous report,^25^ the EZ status had a strong correlation with visual outcomes in the acute phase. However, in this study, evaluation of baseline EZ band defects was challenging because of severe ME and retinal hemorrhage extending into the fovea; thus, these evaluations were excluded. We also compared the rates of NVG and VH among the 4 groups using Fisher exact test.

Missing values were calculated using MissForest methodology^26^; we performed this calculation using the missing package (https://github.com/epsilon-machine/missingpy).

For comparisons between the nonischemic and ischemic categories, the t-test was used for continuous variables, and the chi-square test was used for discrete variables. These specific statistical analyses excluded the 24 eyes that did not undergo FA evaluations at baseline. The level of statistical significance was set to 0.05 for all tests.

## Results

In total, 141 eyes from 141 consecutive patients presenting with unilateral, treatment-naive acute CRVO were evaluated in this study (mean age: 69.3 ± 12.4 years; 88 men and 53 women). Upon initial examination, all eyes (100%) exhibited retinal hemorrhage and ME, with a subset presenting serous retinal detachment at the fovea. From these eyes, 29 received a single initial intravitreal anti-VEGF injection, whereas the remaining 112 underwent a regimen of 3 monthly intravitreal anti-VEGF injections. None of the eyes were subjected to alternative ME treatments such as bevacizumab injection, grid laser photocoagulation, steroid therapy, or surgical intervention.

Out of the 141 eyes enrolled in this study, 117 (83.0%) underwent FA evaluations at baseline. Of these, 13 eyes (11.1%) were diagnosed with ischemic CRVO. The remaining 104 eyes evaluated using FA were identified as having nonischemic CRVO. Furthermore, of the total enrolled eyes, 24 eyes did not undergo FA evaluations, and therefore their ischemic status remained undetermined. With regard to the eyes diagnosed with ischemic CRVO, there were 5 eyes in Group 0, 1 each in Groups 1 and 2, and 6 in Group 3.

Table 1 shows demographic data for all included patients. At baseline, the mean logMAR BCVA was 0.65 ± 0.52 (Snellen equivalent range: 20/20–20/2000) and mean FT was 661.1 ± 257.4 μm. The mean number of anti-VEGF injections administered for CRVO-ME was 5.6 ± 3.6. In total, 112 eyes treated at Saneikai Tsukazaki Hospital, the Kyoto University Graduate School of Medicine, and the Tokushima University Faculty of Medicine initially received 3 monthly injections of aflibercept or ranibizumab, whereas 29 eyes treated at the Kagawa University Faculty of Medicine received a single initial injection of aflibercept or ranibizumab.

**Figure 2:**
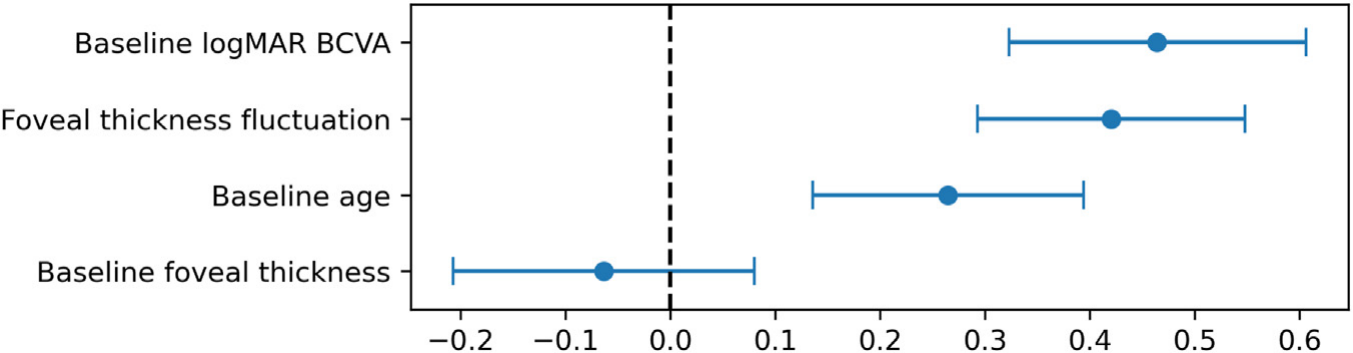
Multiple regression analyses determining the factors associated with the final logarithm of the minimum angle of resolution (logMAR) best-corrected visual acuity (BCVA) in eyes with central retinal vein occlusion receiving long-term anti-VEGF treatment for recurrent macular edema The associated 95% confidence interval (CI) of the baseline logMAR BCVA is 0.32 to 0.61 (*P* < 0.01). The associated 95% CI of the foveal thickness fluctuations is 0.29 to 0.55 (*P* < 0.01). The associated 95% CI of the baseline age is 0.14 to 0.39 (*P* < 0.01). The associated 95% CI of the baseline foveal thickness is −0.21 to 0.08 (*P* = 0.38). The vertical axis shows the explanatory variables and the horizontal axis shows the regression coefficients. The blue lines indicate 95% CIs. We defined explanatory variables, wherein the 95% CIs do not include 0, as statistically significant.

At the 24-month examination, the mean logMAR BCVA and FT were 0.52 ± 0.57 (Snellen equivalent range: 20/13–counting fingers) and 328.9 ± 157.8 μm respectively; these values were significantly lower than the baseline values (*P* < 0.01 for both). We examined the associations of the baseline logMAR BCVA, baseline age, and FTF during the observation period with the final BCVA in order to determine the prognostic factors for the final visual outcome (Fig 2). Multiple regression analysis showed that the final BCVA was not significantly associated with the baseline FT (β = −0.06), with an associated 95% confidence interval of −0.21 to 0.08 (*P* = 0.38). However, age, the baseline logMAR BCVA, and FTF were significantly associated with the final logMAR BCVA (β = 0.26, 0.46, and 0.42, respectively), with associated 95% confidence intervals of 0.14 to 0.39 (*P* < 0.01), 0.32 to 0.61 (*P* < 0.01), and 0.29 to 0.55 (*P* < 0.01), respectively.

### FTF, VA, and Foveal Photoreceptor Status

**Figure 3:**
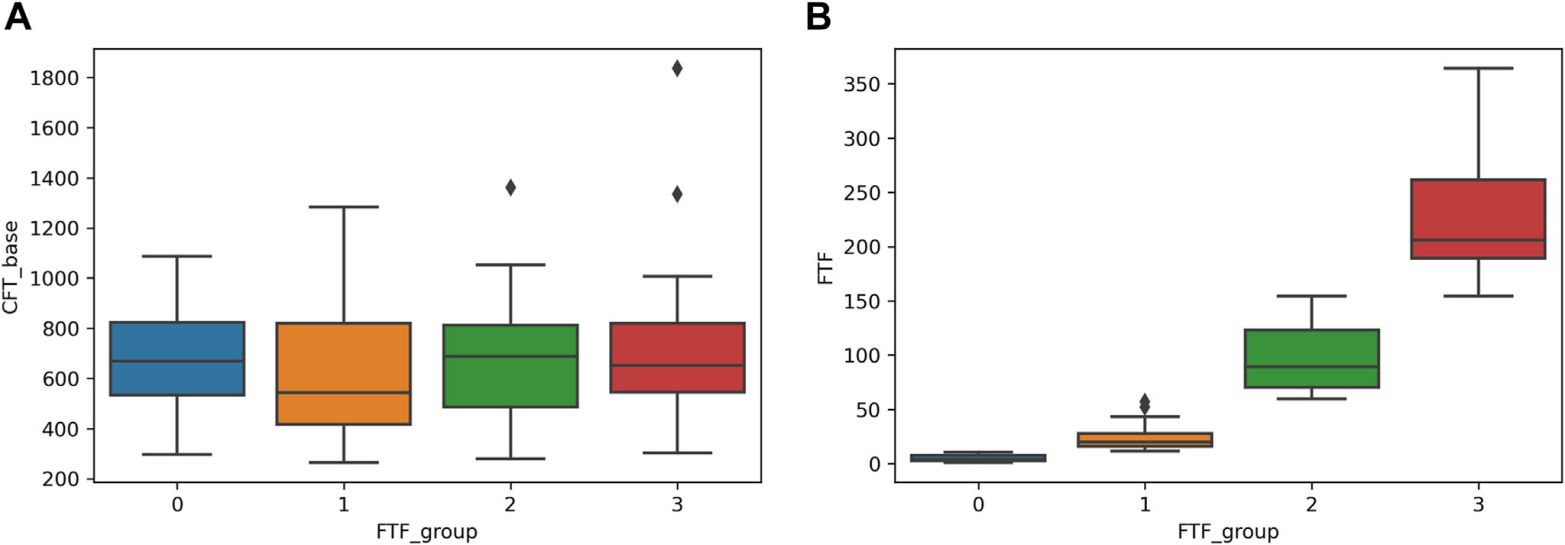
Box plots showing the baseline foveal thickness (FT) and FT fluctuation (FTF) during the observational period (except the baseline visit) in eyes with central retinal vein occlusion (CRVO) receiving long-term anti-VEGF treatment for recurrent macular edema (ME) To evaluate the degree of recurrence of CRVO-associated ME during the observation period, we calculated the standard deviations (SDs) in FT values for each patient based on the FT values recorded at each visit except the baseline visit. We evenly divided the included patients into 4 groups in ascending order of SDs, Group 0 (minimum SD) to Group 3 (maximum SD). The dividing line in each box indicates the median value, the box limits indicate the upper and lower quartiles, and the whiskers indicate the upper 95th and lower fifth percentiles. The black diamonds represent outliers. **A,** Associations between baseline FT and FTF during the observation period. **B,** SD ranges, which were 0 to 10.62, 10.62 to 57.08, 57.08 to 154.24, and 154.24 to 364.07 for Groups 0, 1, 2, and 3, respectively. CFT = central foveal thickness.

We divided the included patients equally into 4 groups (Groups 0–3) in ascending order of the FTF values during the observation period (Fig 3). The baseline FT showed no significant between-group differences (*P* > 0.05 for all). The mean number of anti-VEGF injections administered for CRVO-ME during the follow-up period was 4.4 ± 2.0 in Group 0, 4.8 ± 4.0 in Group 1, 5.4 ± 3.3 in Group 2, and 7.8 ± 3.8 in Group 3 (Fig 3). Thus, the total number of injections was significantly higher in Groups 2 and 3 than in Groups 0 and 1 (*P* < 0.01 for all). Moreover, the visit numbers and the deviations in visit intervals showed no significant difference between groups (*P* > 0.05 for all).

**Figure 4:**
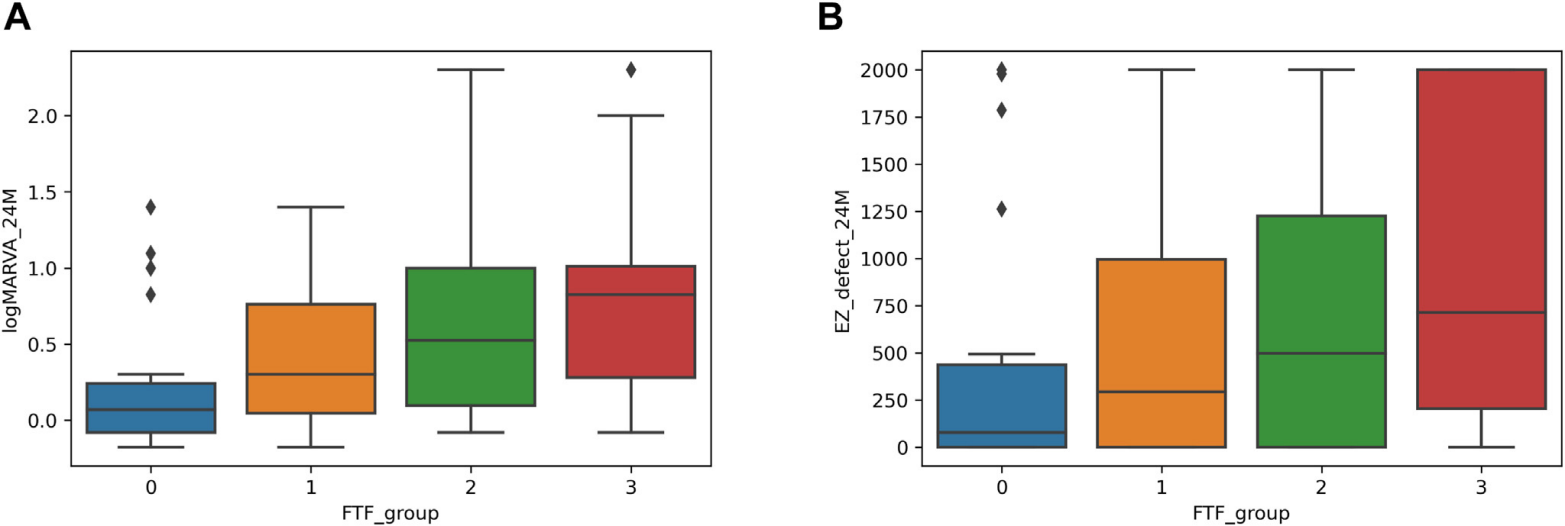
Box plots showing the associations of foveal thickness fluctuation (FTF) with the final logarithm of the minimum angle of resolution (logMAR) best-corrected visual acuity (BCVA) and the length of the foveal ellipsoid zone (EZ) band defect in eyes with central retinal vein occlusion receiving long-term anti-VEGF treatment for recurrent macular edema. The dividing line in each box indicates the median value, the box limits indicate the upper and lower quartiles, and the whiskers indicate the upper 95th and lower fifth percentiles. The black diamonds represent outliers. **A,** Association between FTF and the final logMAR BCVA. **B,** Association between FTF and the length of the final foveal EZ band defect. The final logMAR BCVA is significantly poorer while the final foveal EZ band defect is significantly longer in the group with greater FTF (Group 3) than in the groups with smaller FTF (Groups 0, 1, and 2).

At the final examination, the logMAR BCVA values were 0.22 ± 0.43 (Snellen equivalent range: 20/13–counting fingers) in Group 0, 0.41 ± 0.45 (Snellen equivalent range: 20/15–20/500) in Group 1, 0.61 ± 0.59 (Snellen equivalent range: 20/15–20/2000) in Group 2, and 0.82 ± 0.61 (Snellen equivalent range: 20/20–counting fingers) in Group 3 (Fig 4), while the lengths of the foveal EZ band defects were 381 ± 632 μm, 556 ± 640 μm, 710 ± 715 μm, and 985 ± 838 μm, respectively (Fig 4). Thus, the Group 3 eyes, which exhibited the greatest FTF, showed a significantly longer foveal EZ band defect and a significantly poorer logMAR BCVA than the eyes in the other groups (*P* < 0.01 for all, Fig 4). Foveal thickness fluctuation was significantly and positively associated with logMAR BCVA and the length of the foveal EZ band defect at the final examination.

**Figure 5:**
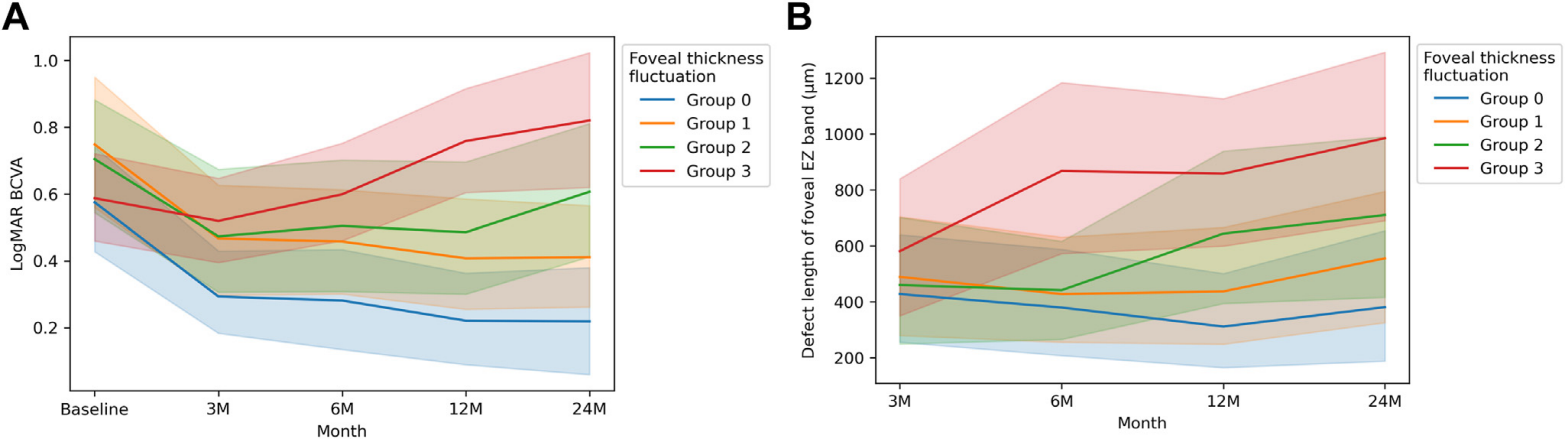
Longitudinal changes in the logarithm of the minimum angle of resolution (logMAR) best-corrected visual acuity (BCVA) and the length of the foveal ellipsoid zone (EZ) band defect according to foveal thickness fluctuation in eyes with central retinal vein occlusion receiving long-term anti-VEGF treatment for recurrent macular edema. **A,** Longitudinally, the logMAR BCVA shows improvement in Groups 0 and 1, remains unchanged in Group 2, and worsens in Group 3. **B,** The foveal EZ band defect length has not increased in Group 0; however, it shows a gradual increase in Groups 1, 2, and 3 (**B**).

Next, we examined longitudinal changes in the logMAR BCVA and length of the foveal EZ band defect in each group (Fig 5). We found that BCVA showed improvements in Groups 0 and 1, remained unchanged in Group 2, and worsened in Group 3. The foveal EZ band defects remained unchanged in Group 0 but gradually became longer in Groups 1, 2, and 3.

### Neovascular Complications

The mean logMAR BCVA of eyes diagnosed with ischemic CRVO and nonischemic CRVO was 0.84 ± 0.53 and 0.55 ± 0.48, respectively, and the difference between them was statistically significant (*P* < 0.05).

**Figure 6:**
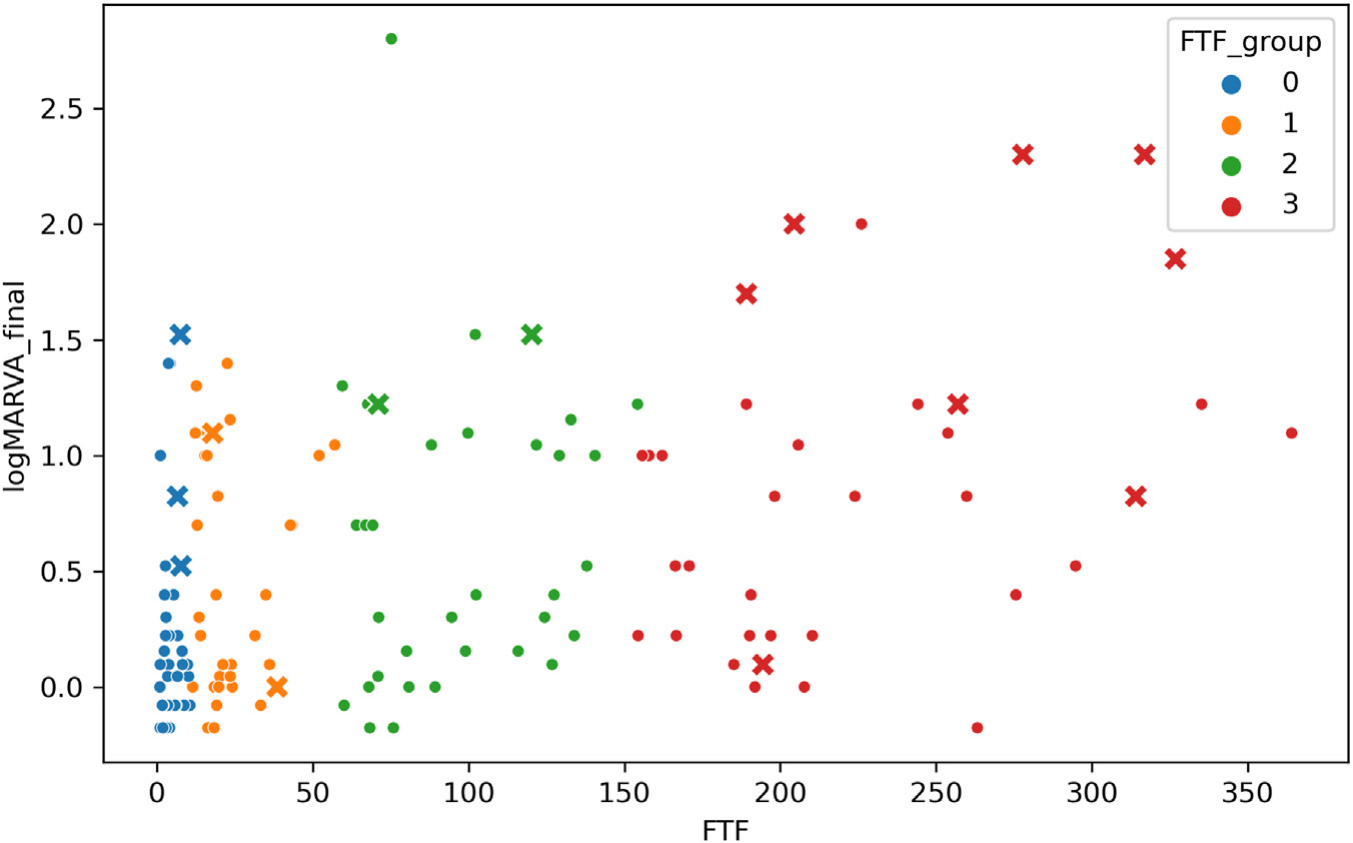
Scatter plots of foveal thickness fluctuation (FTF) and the final logarithm of the minimum angle of resolution (logMAR) best-corrected visual acuity (BCVA) for each eye with central retinal vein occlusion receiving long-term anti-VEGF treatment for recurrent macular edema. The crosses (×) represent eyes that developed neovascular complications during the follow-up period. The final logMAR BCVA in the group with neovascular complications is substantially poorer than that in the group without complications. The neovascular complication rate is not significantly different among groups.

Figure 6 shows the scatter plots of the FTF values and final logMAR BCVA values for each patient and FTF group. The crosses (×) represent eyes with neovascular complications (NVG and/or VH) during the follow-up period. The final logMAR BCVA of patients with neovascular complications was 1.27 ± 0.72, which was substantially poorer than that of patients without complications (*P* < 0.001). The rate of neovascular complications was 8% (3/36 eyes) in Group 0, 6% (2/35 eyes) in Group 1, 6% (2/35 eyes) in Group 2%, and 23% (8/35 eyes) in Group 3, with no significant between-group differences (*P* = 0.106, Fisher exact test).

## Discussion

In this retrospective, multicenter study, we enrolled 141 patients with treatment-naive acute CRVO-ME and examined the 24-month outcomes of anti-VEGF treatment. For each ME recurrence, an additional anti-VEGF injection was administered. An average of 5.6 injections were required during the observation period. The number of anti-VEGF injections required in the present study may be lower than those required in other clinical trials.^27^^,^^28^ However, the final logMAR BCVA was significantly improved, with a decrease in FT relative to the baseline thickness (Table 1). These results suggest that the PRN regimen evaluated in this study was associated with good visual and morphological outcomes, indicating its effectiveness over a long-term follow-up period. In this study, we also enrolled patients with severe CRVO, which may have been responsible for the relatively low number of anti-VEGF injections compared to those in other clinical trials.^4^, ^5^, ^6^, ^7^, ^8^ Previous research has indicated that worse macular ischemia reduces the likelihood of diabetic macular edema.^29^ Similarly, in cases of severe CRVO, the recurrence of macular edema was less frequent, resulting in a lower number of required injections.

Prior studies have demonstrated that among patients with diabetic ME^30^ and patients with BRVO,^18^ those with larger FTF had poorer BCVA. Similarly, among patients with neovascular AMD, those with larger FTF had poorer VA as well as macular fibrosis and atrophy during an observation period of 2 years.^17^ However, the clinical significance of FTF has not been studied well in treatment-naive patients with acute CRVO. Compared to the status of the retinal circulation in eyes with BRVO, that in eyes with CRVO is considered more severely impaired, and impaired circulation involving the macula lowers the likelihood of ME occurrence.^19^

According to the methodology of previous studies,^17^^,^^18^ in the current study, we calculated SDs in FT for each patient using the FT values obtained at each visit (except the baseline visit) in order to evaluate the degree of CRVO-ME recurrence during the observation period (Fig 3). Similar to the results of previous reports involving eyes with diabetic ME, BRVO, and AMD, patients with greater FTF had significantly longer foveal EZ band defects as well as poorer BCVA at the final examination than those with smaller FTF (Fig 4). Moreover, we found that FTF during the observation period longitudinally deteriorated BCVA as well as the foveal photoreceptor status (Fig 5). These results suggest that larger FTF promotes the progression of foveal photoreceptor damage and visual impairment. A shift in the balance between VEGF and connective tissue growth factor has been identified as a predisposing factor in the development of fibrosis.^31^ Intermittent stretch is known to result in the recruitment of macrophages that trigger fibrosis in nonocular tissues.^32^ Therefore, eyes with a larger FTF may have a stronger activation of the vasofibrotic switch by biological mechanisms compared with eyes with a smaller FTF, and this increased activation may be involved in increasing FTF. We believe that the PRN regimen used in this study was useful for the long-term management of most patients with CRVO. However, there might be a need for a more aggressive regimen for eyes with larger FTF. For example, for eyes with diabetic ME,^33^ BRVO,^34^ and AMD,^35^ the efficacies of anti-VEGF treatments based on a treat-and-extend regimen have been reported in several clinical studies.

Gu et al^36^ recently reported weak to moderate associations between changes in FT and changes in BCVA in eyes with CRVO-ME treated with anti-VEGF agents. However, the authors did not examine the association with the final BCVA. Scott et al^37^ reported that greater FTF as assessed by the SD was negatively associated with the VA letter score at month 12. However, in their study, participants with a protocol-defined poor or marginal response at 6 months were switched to alternative treatments. In addition, Chen et al^38^ reported that a larger FTF is associated with poorer visual outcomes in patients with RVO treated with anti-VEGF agents. However, their study included a small number of cases and a short observation period of 12 months. Therefore, we consider our results to be clinically meaningful because a relatively large number of cases were investigated and followed over the course of long-term anti-VEGF agent monotherapy. The usefulness of the treat-and-extend regimen for CRVO-ME as well as the characteristics of patients receiving this or other regimens should be clarified more thoroughly and comprehensively in the future.

In the present study, the visual outcome was strongly associated with FTF during the observation period and the development of neovascular complications. However, the number of patients who developed neovascular complications was not significantly different among groups based on FTF (Fig 6). This finding suggests that the risk of neovascular complications is not strongly associated with FTF, probably because retinal vein occlusion-associated ME is less likely to occur in eyes with poor retinal perfusion.^23^ Our results might be partially consistent with those of a previous study,^24^ because patients with smaller FTF had significantly better final visual outcomes than did those with larger FTF (Fig 4). However, in the present study, the final BCVA of patients with small FTF tended to be severely impaired if neovascular complications occurred (Fig 6). It should be noted that the visual prognosis of eyes with CRVO can be poor if neovascular complications occur, even if FTF is small. The association between small FTF and a poor visual outcome could be a clinical characteristic of eyes with CRVO, but not of eyes with BRVO.

This study had several limitations. First, and most significant, was its retrospective design, because of which the administered anti-VEGF agents varied among patients. Second, the study was conducted in a multicenter setting, and examiners and imaging devices were not standardized; this may have caused a bias in the measurements. Third, some of the enrolled patients with CRVO had systemic and other ocular diseases, which may have affected the results. Fourth, we only measured the FTs at the time when the patients visited each facility. Therefore, the actual status of ME during each interval between visits remains unclear.

Nevertheless, the current study provides a new clinical perspective based on comprehensive evaluations of 2-year outcomes of anti-VEGF treatment with a PRN regimen for the treatment of CRVO-ME. We found that baseline parameters such as age and BCVA were associated with the visual outcome and foveal photoreceptor status. Moreover, FTF during the follow-up period played a role in longitudinal changes in these parameters and their final values. However, small FTF can also be observed when the retinal circulation involving the macula is poor. In such cases, neovascular complications may occur, resulting in significantly poor visual outcomes. We may be able to improve the visual prognosis of eyes with CRVO by identifying the characteristics of eyes with larger FTF and those with a risk of neovascular complications, as this will permit more strict control of FTF or appropriate measures to reduce the risk of such complications. However, highly-powered prospective studies are warranted to confirm the results of this investigation.

## Data Statement

Daisuke Nagasato, Yuki Muraoka, Naomi Nishigori, Rie Osaka, Yoshinori Mitamura had full access to all study data and take responsibility for the integrity of the data and the accuracy of the data analysis.
